# On the Use of the Stethoscope in Pulmonary Affections

**Published:** 1829-10-01

**Authors:** 


### CLINICAL REVIEW.

### XXXII.

### WORCESTER INFIRMARY.

On the Use of the Stethoscope in Pulmonary Affections. By Chas. Hastings, M.D.

Were auscultation in danger, we should have felt some alarm at the opening of Dr. Hastings' paper :—" Save me from my friends"—alluding to those who run mad after the stethoscope, or expect every thing from it, without the trouble of other modes of investigation. Those who do so are very inexperienced and very unwise. The diagnosis of diseases is the most difficult branch of our science, and he who expects to render it perfect and easy, even in diseases of the lungs, by the aid of the stethoscope, will be grievously disappointed. We are well aware that odium is daily brought upon this auxiliary by imprudent professors of stethoscopy in this country. But what remedy, in therapeutics, has not been abused on its first introduction into practice ?

Dr. Hastings cannot except even Laennec, " from the imputation of attaching too much importance to the information to be derived from this source (auscultation.) He obviously inclines to rest his dependence on the results to be derived from the use of auscultation and percussion, *to the exclusion of the important aid to be procured from a diligent study of the symptoms*." We think this last part of the sentence is too severe; for we no where in Laennec's work see any evidence of this exclusion of symptomatology. Turn to any section of that great pathologist's treatise—emphysema of the lungs, for example (being the first place we opened in the volume before us)—and we see that the author first describes the anatomical characters, in other words, the morbid anatomy of the disease—then the occasional causes—thirdly, the symptoms of the disease, independently of auscultation—and, fourthly, the physical signs afforded by the stethoscope. This is the course generally pursued by Laennec, and what better course could be pursued ? Where is the ground for accusing him of excluding symptomatology in such procedure ? We agree, indeed with Dr. Hastings, that—

" If such a divorce of these helpmates to each other were ever to happen, the discovery of the stethoscope would become comparatively of no avail, for it is only by a proper consideration of the knowledge to be procured from each of these sources, that a correct judgment can be formed of obscure pulmonary affections."*

It is true that Laennec may have been deceived in his opinion that, in pectoriloquism, he had discovered a " true pathognomonic sign" of phthisis pulmonalis. This is quite another question.

" Laennec," says our author, " considered that he had, in pectoriloquism, discovered a true pathognomonic sign of phthisis pulmonalis. The only sign to be regarded as certain. There can be no doubt of its being a sign of great importance, for this phenomenon is invariably observed when the excavated end of the instrument is applied over a cavity of the lungs communicating with the trachea by means of a bronchial ramification, and the person to whom it is applied, pronounces audibly a given number of syllables. Thus far, then, we may predicate with safety, that where pectoriloquism exists, there is a corresponding cavity in that part of the lungs; and also that pectoriloquism is a pathognomonic sign of phthisis, if the existence of an excavation in the lungs is necessarily accompanied by consumption; *or if pulmonary consumption can never happen without the occurrence of a tubercular excavation : but I am prepared to argue that excavation of the lungs may exist without the coexis-*

* Midland Reporter, No. V. p. 235.

*tence of phthisis; and also that tubercular phthisis very commonly happens, unattended by any excavation, and consequently that pectoriloquism is not a pathognomonic sign of pulmonary consumption.*

"We may first enquire, does the existence of a tubercular excavation necessarily imply the coexistence of phthisis? That, in infinitely the greater number of persons thus affected, consumptive symptoms also occur, the history of this disease unfortunately, but too decidedly, testifies. But are there not, I would inquire, individuals in whom the existence of such a cavity is consistent with tolerable health. In whom, by length of time, the excavation is emptied of all tuberculous or purulent matter, and becomes lined with a smooth polished membrane? Such persons regain their flesh, and sometimes enjoy years of health, and consequently cannot be said to be affected with phthisis, although, if we consider pectoriloquism a certain sign of phthisis, they must be considered to be so. Laennec has himself described cases of this kind, and other authors have confirmed his observations."

In the first place we may observe, that Laennec merely gives it as his opinion, that *tuberculous* phthisis is the only kind of phthisis which we should admit, "unless, indeed, it were the *phthisis nervosa,* and the chronic catarrh simulating tuberculous phthisis." As to the impossibility of phthisis occurring without an excavation, Laennec has made no such assertion. On the contrary, he observes:—"When there exists a great number of tubercles, even very small ones, in the lungs, death will sometimes take place *before any* of them has reached such a degree of softness as to have their contents discharged into the bronchia, and consequently to leave any ulcerous excavation." As to recovery after excavations, it does not prove that the disease was not tubercular phthisis, but only that the excavations will sometimes heal. We cannot, upon the whole, but think that Dr. Hastings has set up a man of straw himself, which any body could have knocked down with a feather.

Leaving these discussions aside, the cases which Dr. Hastings has published in this paper are interesting in themselves.

*Case* 1. This was a man, 70 years of age, who died in the Worcester Infirmary of ulceration of the pyloric orifice of the stomach. He said he had nearly died of consumption in his youth. On dissection, a tuberculous excavation, capable of holding a walnut, was found in the superior lobe of the right lung, lined with a polished membrane. There were several hard tubercular masses in the lungs generally.

*Case* 2. This was also an elderly person, who had, in early life, been subject to cough. She died of fever. In the left lung, there was a cavity, with a smooth polished lining, and some hard tuberculous masses dispersed through the lungs.

It is well known that dilated bronchial tubes will exhibit the same auscultic phenomena as excavations, and, consequently, the existence of pectoriloquism, *per se,* does not necessarily prove that the patient is affected with tubercular phthisis and excavations.

"It is also not very uncommon to meet with instances of consumption, where the disease is of much more rapid course, owing to the conjunction of inflammation of the cellular or bronchial tissue of the organ, with numberless miliary tubercles scattered throughout the lungs, and in some parts coalescing, and forming large tuberculous masses. In such instances, death often seizes its victim ere any excavation has formed, and of course before pectoriloquism can inform us of the tuberculous deposit. But although the cylinder, in such instances, does not offer us what Laennec states to be the certain sign, it is, under such circumstances, often exceedingly useful in pointing out that much mischief is progressing in the lungs. For the absence of the usual respiratory murmur in certain parts of the lungs, where it ought to exist, may justly lead us to pronounce, after sundry trials of the fact, that there exists some unnatural production in the substance of the lungs. In this way, the attentive auscultator may be enabled to form an opinion of the probable termination of consumption in its more early stages, long before excavations are formed, or the symptoms manifest any severe lesion of this important viscus. That such knowledge is of the first consequence to the practical physician, no sensible man will, I presume, deny. It may, in some cases, by pointing out the necessity of early treatment, enable us to avert the fatal issue of a disease, whose ravages annually sweep off a large portion of the population of Great Britain. I am certainly, from some experience, decidedly of opinion, that if our treatment of tuberculous consumption were more early and assiduously applied, we should not so often have to deplore the inefficiency of our art in this dreadful malady."

The following case is adduced by our author as an instance of pulmonary consumption checked, in its early stage, by appropriate treatment.

*Case* 3. A young female, aged 17, was received into the Worcester Infirmary in the month of September, affected with dyspnœa on slight exertion, and short dry cough. Pulse was 100 and small—tongue clean—bowels regular.

"By the stethoscope, the respiratory murmur was found to be puerile over the whole of the right side of the chest. On the left side, in several parts, it was puerile also, but towards the upper part of that side, there was a spot more than an inch square, in which no respiratory murmur could be heard. Repeated examination with the cylinder confirmed this result. I accordingly, comparing the information derived from auscultation with the general state of health, concluded, that a portion of the lung on that side, was rendered impermeable to air, by the deposition of tuberculous matter."

Dr. H. ordered a seton to be inserted over the part. She was bled, and took large quantities of decoction of sarsa, with Brandish's liquor potassæ, for a considerable time.

"It gave me much gratification to observe, that the plan succeeded in amending her general health. After remaining in the infirmary some time, she was discharged in tolerable health; but the respiratory murmur had not then returned in the spot before alluded to. I saw her two years afterwards, looking pretty well, but I had not an opportunity of examining whether there was still an absence of the respiratory murmur in the affected part."

No one will accuse us of undervaluing the benefits of auscultation; but we were not prepared for its powers in discriminating such a case as the one just stated. The want of respiratory murmur in a square inch of lung, on one side, appears to us a sorry proof of incipient phthisis. And the cure of this incipient phthisis by a seton, venesection, and sarsaparilla, *the want of respiratory murmur continuing when the patient was discharged in "tolerable health,"* is really a species of medical reasoning which we did not expect from Dr. Hastings. We think that, on reflection, the highly talented author will acknowledge that he has fallen into the error of which he has accused Laennec—that of trusting too much to auscultation in the diagnosis of pulmonary diseases. We are not aware that the stethoscope can be depended on for the detection of a deposition of tuberculous matter to the extent above-mentioned, or, indeed, to any extent. Whoever has been in the habit of employing this instrument frequently will acknowledge this. In the foregoing case, the constitutional symptoms were by no means so decided as to authorise the conclusion that incipient phthisis obtained—and the auscultic sign remained after the symptoms were removed.

*Case* 4. This case is related with the view of shewing that the stethoscope failed to shew the state of lung that was present when the examinations were made.

"The subject of this curious affection was 66 years of age. He was of a thin spare habit, and had been ill with a cough for several years. He had, in fact, been considered asthmatical for five years before I saw him. During the whole of that period, he had rather lost flesh, and in each succeeding winter and spring, had suffered a considerable aggravation of cough, expectoration, and difficulty of breathing. I was first called to see him in March, 1827. He then complained of distressing cough, particularly at night, and he could not lie down in bed comfortably, for he no sooner attempted it than he was called up by cough. The expectoration was very various, but for the most part frothy; sometimes the frothy matter was mixed with a small portion of pus-like fluid; sometimes the pus-like matter was in great quantity, and occasionally, a tough mucus tinged with blood, was, after a severe fit of coughing, expectorated. The cough came on usually in fits, and was deep and sounding. The pulse was 80; tongue loaded; stools unnatural; urine pale and abundant; the breathing was laborious, and the muscles of the chest were called into unnatural action.

"By percussion, the chest gave a louder than the natural sound, both on the right and left side. The stethoscope discovered an audible rattle in several parts of the chest. It was a wheezing noise, and did not resemble the mucous rattle of bronchitis; in other parts of the chest, scarcely any rattle or respiratory murmur could be heard. I could not, in any part of the chest, discover pectoriloquism. The result of this examination did not much improve my knowledge of the disease of my patient, and it certainly did not lead me to expect that tuberculous disease had proceeded to any great extent. The result of percussion and auscultation, rather favoured the notion of emphysema, but the state of the symptoms was not explained by that view of the case. He lived about a month afterwards, and his death afforded me an opportunity of investigating the condition of the thoracic viscera.

"On opening the chest, the lungs appeared larger than usual. On examining them more accurately, the air vessels under the pleura were found, for the most part, very much enlarged, and this gave the apparent increase of magnitude to the lungs. On pressing the lungs, the displacement of air was heard, not at all like the ordinary pulmonary crepitus. The pleuræ were not adherent. When the lungs were cut, air escaped, and then there came in sight numberless miliary tubercles, imbedded in the pulmonary tissue. By extending the examination over each of the lungs, the tuberculous bodies were every where found numerous; but in the superior lobes of each lung, they were particularly so, and in several parts they coalesced. The cellular tissue was rather inflamed and thickened. The mucous membrane of the bronchia was much inflamed and thickened. There was considerable dilatation of the bronchial vessels.

"The abdominal viscera were healthy, excepting the liver, in which organ there was a deposition of tuberculous matter."

In this case, our author observes, the stethoscope and percussion failed to give warning of the existence of tuberculous degeneration, on account of the very rare conjunction of extensive emphysema of tbe lungs with miliary tubercle. The following very remarkable and melancholy case concludes Dr. Hastings' paper.

*Case* 5. "On the 19th of August, 1825, I was consulted respecting a young lady, who had been for two months in a dubious state of health, from a very slight cough, with trifling expectoration in the morning, which had once or twice been tinged with blood. On the morning of my visit, she had expectorated some blood, unmixed with pus or mucus, and of a scarlet colour. Her parents were, on this account, under great alarm, as they had previously lost three children in consumption. There was nothing, however, in the general appearance of the young lady, to have given alarm to an ordinary spectator. She was rather full of flesh, and considered that she had rather gained than lost in that particular during the last two months. To an experienced eye, however, she had not a healthy look, for the countenance was rather bordering on the cadaverous, and the eyes were dull. The hands felt clammy, and there was an unaccountable listlessness in the young lady's manner. The pulse was 76; the tongue was clean, and the bowels were regular. I could not trace any feverish action during the twenty-four hours. The breathing was free, and a full inspiration caused no uneasiness. The catamenia were regular. In short, she declared, that excepting the slight barking cough which attacked her in a morning, when at the toilette, she was in perfect health. She betrayed, nevertheless, much perturbation at the sight of the expectorated blood, and declared a firm conviction that the disease was similar to that of which her relations had died.

"I examined, at this visit, the chest very particularly, and found that percussion produced a natural sound on each side. With the stethoscope, the respiratory murmur was heard natural on the right side, and no indication of any excavation of the organ. On the left side of the chest, however, perfect pectoriloquism was heard, on the front of the chest, between the second and third, and third and fourth ribs.

"Some blood was taken from between the shoulders by the cupping-glasses, and I directed a mixture of infusion of roses and tincture of digitalis, to be taken every four hours. I also advised quietude and a milk diet.

"In a few days, the spitting of blood disappeared, and this amiable young lady described herself in perfect health again, excepting that she had a slight morning cough, and trifling expectoration. She walked about, without any hurry of respiration, and seemed anxious not to be considered an invalid. It was notwithstanding, but too evident, that the disease was formidable, for another examination with the stethoscope established still more strongly my conviction, that there existed an excavation in the superior lobe of the left lung. The eyes also looked peculiarly languid, and the hands had a deadly clammy feel.

"She went on with very little appearance of illness, and without losing any flesh, till the 15th of September, on the morning of which day, she coughed up about two tea-spoonfuls of dark-coloured blood, accompanied with a small quantity of pus-like matter. The pulse was 94. The breathing was not at all hurried, and there was not any heat of the skin. By appropriate treatment, this bleeding subsided, and did not return till the 28th of the same month, when a sudden exasperation of the symptoms took place. The breathing became hurried; the cough much more troublesome, and a considerable quantity of scarlet-coloured blood was expectorated. Various means were ineffectually tried to arrest the progress of the disease. The bleeding repeatedly occurred, and my patient died in the morning of the 8th of October.

"*Examination of the body*. No considerable emaciation had taken place. On elevating the sternum, nothing worthy of remark presented itself. On removing the lungs, they were found slightly adherent on the right side, but much more so on the left; on both sides the adhesion was high up, and in the direction of the axillæ.

"The internal surface of the trachea was free from purulent secretion. The bronchia contained a small quantity of sero-sanguinolent fluid. On tracing the ramifications in the right lobes, the only appearances were a rather suffused state of the blood-vessels in some parts; the lungs of a soft structure, and the air-vessels throughout, pervious.

"The general appearances of the left lobes resembled those of the right; but on the outer and upper surface of the superior lobe, appeared a cavity large enough to admit the half of a middle-sized orange, which had been prevented from opening into the sac of the pleura, by the adhesion of the pulmonary to the costal pleura. This cavity communicated freely with the bronchial tubes. Pus was searched for, but the slightest trace of it could not be found; nor was there the slightest degree of hardness in the sides of this ulcer, indicative of the process of adhesive inflammation having been set up. On the contrary, the appearance was that of simple ulcerative absorption rapidly advancing."

Dr. Hastings very properly observes, that these failures do not warrant our rejection of auscultation. "For there is scarcely an affection of any one of those organs (thoracic) which may not be, in some degree, made clear, by studying the physical signs that are afforded by auscultation and percussion."

We return Dr. Hastings sincere thanks for the interesting cases which he has here most candidly laid before his professional brethren. Would that all of them were as modest and judicious as the talented writer of the foregoing communication.

---

